# Descriptive study of the Specialized Care of the Spanish Health System

**DOI:** 10.11606/S1518-8787.2018052000289

**Published:** 2018-01-29

**Authors:** Karen Nombela-Monterroso, Víctor M González-Chordá, Pablo Roman

**Affiliations:** I Universidad Jaume I. Departamento de Enfermería. Castellón, España; II Universidad de Almería. Departamento de Enfermería, Fisioterapia y Medicina. Almería, España

**Keywords:** Health Services, Tertiary Healthcare, Quality Indicators, Health Care, trends, Health Care Quality, Access, and Evaluation, Ecological Studies, Servicios de Salud, Atención Terciaria de Salud, Indicadores de Calidad de la Atención de Salud, tendencias, Calidad, Acceso y Evaluación de la Atención de Salud, Estudios Ecológicos

## Abstract

**OBJECTIVE:**

The objective of this study is to analyze the trend of the Key Indicators of the National Health System of Spain and its autonomous communities, related to Specialized Care, from the publication of the Law of Cohesion and Quality.

**METHODS:**

This is an ecological study of temporary series of Spain and its autonomous communities from 2003 to 2014. We have analyzed 10 indicators related to Specialized Care (percentage of expenditure, professionals, waiting lists, surgical activity, average duration, infections, and mortality) using the Prais-Winsten regression method. We have obtained data from the health information system of the Spanish Ministry of Health, Social Services, and Equality.

**RESULTS:**

Specialized care expenditure (APC = 0.059, 95%CI 0.041–0.074), number of medical professionals (APC = 0.0006, 95%CI 0.0003–0.0009) and nursing professionals (APC = 0.001, 95%CI 0.0005–0.0016), hospital infections (APC = 0.0003, 95%CI 0.0002–0.0004), and in-hospital mortality (APC = 0.0008, 95%CI 0.0006–0.001) had an increasing trend in Spain. Average duration presented a decreasing trend (APC = -0.0017, 95%CI -0.002– -0.0014). The trend of waiting lists (specialized appointment and non-urgent surgical interventions) was static. The trend of these indicators varied in the Autonomous Communities.

**CONCLUSIONS:**

We have observed a non-compliance with the principles of equity and quality of the services offered. Increased aging, technological development, and inadequate strategies taken to reduce health costs may be the main causes.

## RESUMEN

**OBJETIVO:**

Analizar la tendencia de los Indicadores Clave del Sistema Nacional de Salud de España y sus comunidades autónomas, relacionados con la Atención Especializada, desde la publicación de la Ley de Cohesión y Calidad.

**MÉTODOS:**

Estudio ecológico de series temporales de España y sus comunidades autónomas desde 2003 hasta 2014. Se analizaron 10 indicadores relacionados con Atención Especializada (porcentaje de gasto, profesionales, listas de espera, actividad quirúrgica, estancia media, infecciones y mortalidad) utilizando el método de auto-regresión de Prais-Winsten. Los datos se obtuvieron del sistema de información sanitaria del Ministerio de Sanidad, Servicios Sociales e Igualdad español.

**RESULTADOS:**

Existió una tendencia creciente del gasto en atención especializada (APC = 0,059; IC95% 0,041–0,074), número de profesionales de medicina (APC = 0,0006; IC95% 0,0003–0,0009) y enfermería (APC = 0,001; IC95% 0,0005–0,0016), infecciones hospitalarias (APC = 0,0003; IC95% 0,0002–0,0004) y mortalidad intrahospitalaria (APC = 0,0008; IC95% 0,0006–0,001) en España. La estancia media presentó una tendencia decreciente (APC = -0,0017; IC95% -0,002– -0,0014). La tendencia de las listas de espera (consulta especializada e intervenciones quirúrgicas no urgentes) fue estática. La tendencia de estos indicadores varió en las Comunidades Autónomas.

**CONCLUSIONES:**

Se observa un incumplimiento de los principios de equidad y calidad de las prestaciones ofrecidas. El aumento del envejecimiento, el desarrollo tecnológico y estrategias poco adecuadas tomadas para reducir los costes sanitarios pueden ser las principales causas.

## INTRODUCTION

A health system aims to improve the health status of its population with curative, preventive, and rehabilitative care and it is influenced by the political, social, cultural, and economic context of the country in which it is developed^1^. There are different models of health systems, among them, the National Health System (SNS), currently existing in Spain related to the General Health Law of 1986^2^. Its main characteristics are the right of every citizen to health protection, public financing, and provision of services that ensure the quality of care, among others^1^ ^–^ ^3^.

On the other hand, among the principles and criteria of this law, we can find the decentralization of health in Autonomous Communities (AC), granting to each one the responsibility of meeting the health of its citizens^3^. The decentralization process, finalized in 2001, had the objective of adapting the health management to the territorial and demographic characteristics of each AC^4^. In 2003, the Law of Cohesion and Quality of the SNS (LCC-SNS)^5^ was published to ensure citizen participation, quality, and equity of the health care throughout the country. This law understands equity as the right to health protection in conditions of effective equality throughout the territory that allows the free movement of all citizens^1^.

Decentralization processes are justified by advantages such as the adequacy of services to the needs of each territory, the approximation of institutions to citizens or the development of alternative management models, and a source of learning. Thirteen years after the publication of the LCC-SNS, the lack of health equity is one of the main problems deriving from decentralization, especially regarding expenditure and financing, health status of citizens, access to health care, and use of resources^6^.

The LCC-SNS revealed the importance of professionals in the quality of the system and research in the development and effectiveness of health services. It also highlighted the need to address the most prevalent health problems with the elaboration of strategic plans that determine minimum care standards and basic care models, while ensuring the sustainability of the SNS^1^ ^,^ ^5^ ^,^ ^7^. The Royal Decree-Law 16/2012, of urgent measures to ensure the sustainability of the SNS and to improve the quality and safety of its services^8^, was published to address the serious economic situation and the increase in health expenditure, although its application caused economic austerity with significant repercussions in the SNS^9^.

On the other hand, the LCC-SNS entrusts to the Ministry of Health the creation of a Health Information System to ensure the availability of information and communication between the Administration of the Government and the AC^7^.

In this system, we can find the “Key Indicators of the SNS”, which provide, in an integrated and systematic way, data on health expenditure, use of resources, accessibility to services, quality of care, and level of health of the population. This allows us to objectify a determined health situation, evaluate its behavior over time, and know if the proposed objectives and the expected results are achieved to establish improvement actions^10^.

The objective of this work was to analyze the evolution of the Key Indicators of the SNS of Spain and its AC, related to Specialized Care (SC) after the Law of Cohesion and Quality.

## METHODS

This is an ecological study of time series that analyzed the trend of the Key Indicators of the SNS of Spain and its AC, using the Health Information System of the Ministry of Health, Social Services, and Equality related to SC^11^.

We selected indicators that contributed with more information related to the objective of the study, considering as inclusion criteria the availability of the data and the utility to assess trends and possible differences regarding equity and quality of care (Box).

Box **List of selected indicators.**

**Table:** 

Indicators	Formula
Percentage of expenditure on specialized care services	(Public health expenditure in hospital and specialized services / Total current public health expenditure) x 100
Nursing staff in specialized care per 1,000 inhabitants	(Number of nurses / Population) x 1,000
Specialized medical staff per 1,000 inhabitants	(Number of physicians / Population) x 1,000
Patients waiting for specialized care appointments	(Number of patients pending an initial appointment in Specialized Care / Population) x 1,000
Working operating rooms per 100,000 inhabitants	(Number of working operating rooms / Population) x 100,000
Surgical interventions per 1,000 inhabitants	(Total number of surgeries performed in one year / Population in that year) x 1.000
Patients waiting for non-urgent surgical interventions per 1,000 inhabitants	(Number of patients pending a non-urgent surgical intervention / Population) x 1,000
Average duration	Average days of stay of the valid total discharges
Rate of hospital infection	(Number of hospital discharges with nosocomial infection diagnosis in one year / Total discharges in that year) x 100
Global in-hospital mortality per 100 hospital discharges	(Total number of hospital discharges per death / Total discharge) x 100

We included data from the indicators since 2003, which is when the LCC-SNS came into force, until 2014, the last year with information available in the Health Information System^11^ for the selected indicators.

Trend estimation was based on the calculation of annual percentage change (APC) and its 95% confidence intervals (95%CI), using the Prais-Wisnten regression method for the analysis of time series, which allows us to obtain an overall trend for a given period^12^. The analysis was performed on all indicators for the SNS and each AC using the program Stata 14.0 (2015).

## RESULTS

The percentage of expenditure dedicated to SC in Spain increased by 9.5 points between 2003 and 2014, from 46.3% to 55.8%, with an increasing trend (APC = 0.059, 95%CI 0.041–0.074). We observed an increasing trend in expenditure dedicated to this level of care in thirteen AC, while the trend was static in four AC (Cantabria, Aragon, Balearic Islands, and Madrid). Catalonia was the AC with the greatest increase in SC expenditure (APC = 1.367, 95%CI 0.319–5.731). We observed no decrease of the indicator in any AC, although information for this indicator for Ceuta and Melilla was not available.

The number of nursing and medical professionals per 1,000 inhabitants in Spain showed an increasing trend for nursing (APC = 0.001, 95%CI 0.0005–0.0016) and medical professionals (APC = 0.0006, IC95% 0.0003–0.0009). Melilla presented a static trend in both indicators and Ceuta had a decreasing trend in the number of physicians (APC = -0.0013, 95%CI -0.0013– -0.0001) (Table 1).

**Table 1:** Nursing and medical staff in specialized care per 1,000 inhabitants. Spain, 2003–2014.

AC		2003	2014	APC	95%CI	Trend
Basque Country	Nursing	2.46	4.42	0.0055	0.0031–0.0082	Increasing^b^
	Medical	1.37	2.213	0.002	0.001–0.003	Increasing^a^
Asturias	Nursing	2.76	3.61	0.0018	0.0012–0.0025	Increasing^b^
	Medical	1.75	2.15	0.0009	0.0004–0.0014	Increasing^a^
Aragon	Nursing	3.29	4.3	0.0022	0.0015–0.0029	Increasing^b^
	Medical	1.96	2.15	0.0004	< 0.0001–0.012	Static
Galicia	Nursing	2.62	3.16	0.0012	0.0002–0.0023	Increasing^a^
	Medical	1.52	1.76	0.0005	0.0001–0.0009	Increasing^a^
La Mancha	Nursing	2.40	2.79	0.0009	-0.0002–0.0022	Static
	Medical	1.44	1.74	0.0007	-0.0001–0.001	Static
Castile and León	Nursing	2.68	3.14	0.001	0.0003–0.0018	Increasing^a^
	Medical	1.48	1.86	0.0009	0.0006–0.0012	Increasing^b^
Catalonia	Nursing	2.68	3.16	0.0011	0.0004–0.0018	Increasing^a^
	Medical	1.80	1.84	> -0.0001	-0.006–0.0001	Static
Madrid	Nursing	2.86	3.32	0.001	0.0002–0.0019	Increasing
	Medical	1.55	2	0.011	0.007–0.001	Increasing^b^
Murcia	Nursing	2.49	2.93	0.001	0.0001–0.002	Increasing^a^
	Medical	1.43	1.75	0.0008	0.0004–0.0011	Increasing^b^
Spain	Nursing	2.67	3.14	0.001	0.0005–0.0016	Increasing^a^
	Medical	1.54	1.81	0.0006	0.0003–0.0009	Increasing^a^
Canary Islands	Nursing	2.64	3.07	0.0009	0.0004–0.0014	Increasing^a^
	Medical	1.55	1.78	0.0005	0.0004–0.0006	Increasing^a^
Balearic Islands	Nursing	3.23	3.6	0.0006	-0.0003–0.0017	Static
	Medical	1.60	1.87	0.0066	0.0001–0.001	Increasing^a^
Navarre	Nursing	4.03	4.22	0.0005	-0.001–0.0024	Static
	Medical	2.07	2.39	0.0007	0.0001–0.0013	Increasing^a^
Andalusia	Nursing	2.46	2.61	0.0005	-0.0001–0.001	Static
	Medical	1.30	1.53	0.0005	0.0003–0.0007	Increasing^b^
Extremadura	Nursing	2.62	2.92	0.0006	0.0003–0.0009	Increasing
	Medical	1.45	1.75	0.0006	0.0004–0.0009	Increasing^b^
La Rioja	Nursing	2.88	3.14	0.0003	-0.0005–0.001	Static
	Medical	1.44	1.68	0.0005	< 0.0001–0.0008	Increasing^a^
Valencia	Nursing	2.54	2.64	0.0004	0.0002–0.0006	Increasing^b^
	Medical	1.48	1.648	0.006	< 0.001–0.011	Increasing^a^
Cantabria	Nursing	3.12	3.23	0.0002	-0.0003–0.0007	Static
	Medical	1.61	1.76	0.0002	-0.0002–0.009	Static
Melilla	Nursing	2.72	2.33	-0.0007	-0.0016–0.0003	Static
	Medical	1.32	1.26	-0.0001	-0.0004–0.0002	Static
Ceuta	Nursing	2.93	2.53	-0.0009	-0.0019–0.0001	Static
	Medical	1.76	1.47	-0.0007	-0.0013– -0.0001	Decreasing^a^

AC: autonomous community; APC: annual percent change

a Significance level p < 0.05.

b Significance level p < 0.001.

The rate of patients on waiting lists for specialized appointments (APC = 0.001, 95%CI -0.006–0.008) and the number of patients waiting for non-urgent surgical interventions (APC = 0.001, 95%CI -0.001–0.003) showed a static trend in both cases for Spain. The analysis of the evolution of these indicators from the AC could not be performed because the data were not available.

Working operating rooms reached a total of 9.4 per 100,000 inhabitants in Spain in 2014, with an increasing trend (APC = 0.002, 95%CI 0.001–0.003). Navarre (APC = -0.0008, 95%CI -0.001– -0.0001), Ceuta (APC = -0.003, 95%CI -0.004– -0.001), and Melilla (APC = -0.005, 95%CI -0.007– -0.005) showed a decreasing trend, while 11 AC had an increasing trend and five remained static (Valencia, Castile and León, Canary Islands, Balearic Islands, and Cantabria).

Surgical interventions in Spain showed an increasing trend in the period studied, going from 95.2 to 107.6 interventions per 1,000 inhabitants (APC = 0.292, 95%CI 0.047–1.589). This rate kept an increasing trend in 10 AC and a static trend in six AC (Balearic Islands, Murcia, La Mancha, Andalusia, Navarre, and Melilla). This indicator showed a decreasing trend in the Canary Islands (APC = -0.006, 95%CI -0.008– -0.001), Ceuta (APC = -0.009, 95%CI -0.01– -0.008), and Cantabria (APC = -0.009, 95%CI -0.01– -0.009).

The average stay of patients in 2014 (Table 2) was 7.6 days in Spain, with a decreasing trend (APC = -0.0017, 95%CI -0.002– -0.001). This indicator has declined in recent years in all AC, although the trend can be considered as static in four of them.

**Table 2:** Indicator: average duration in descending order. Spain, 2003–2014.

AC	2003	2014	APC	95%CI	Trend
Ceuta	5.73	5.55	-0.0004	-0.0015–0.0008	Static
La Mancha	7.27	7.1	-0.0005	-0.001–0.0001	Static
Castile and León	7.49	7.2	-0.0005	-0.001–0.0001	Static
Murcia	6.89	6.54	-0.0007	-0.0018–0.0005	Static
Navarre	7.12	6.77	-0.0011	-0.0017–-0.0003	Decreasing^a^
Balearic Islands	7.11	6.63	-0.0011	-0.0015– -0.0006	Decreasing^a^
Extremadura	7.16	6.4	-0.0014	-0.0017– -0.0012	Decreasing^b^
Aragon	7.86	7.14	-0.0015	-0.0023– -0.0007	Decreasing^a^
Catalonia	6.94	6.22	-0.0015	-0.0019– -0.0011	Decreasing^b^
Asturias	8.64	7.71	-0.0017	-0.0021– -0.0013	Decreasing^b^
Spain	7.64	6.83	-0.0017	-0.0020– -0.0014	Decreasing^b^
Andalusia	7.81	6.9	-0.0018	-0.0021– -0.0015	Decreasing^b^
Canary Islands	9.21	8.36	-0.0019	-0.003– -0.0006	Decreasing^a^
Basque Country	7.22	6.46	-0.0019	-0.0028– -0.0008	Decreasing^a^
Valencia	6.70	5.86	-0.0019	-0.0022– -0.0015	Decreasing^b^
Galicia	8.99	7.94	-0.002	-0.003– -0.0008	Decreasing^a^
La Rioja	6.95	5.8	-0.0021	-0.0027– -0.0015	Decreasing^b^
Madrid	8.64	7.27	-0.0025	-0.003– -0.0021	Decreasing^b^
Cantabria	8.17	6.84	-0.0026	-0.0033– -0.0019	Decreasing^b^
Melilla	6.88	5.56	-0.003	-0.0039– -0.0008	Decreasing^b^

AC: autonomous community; APC: annual percent change

a Significance level p < 0.05.

b Significance level p < 0.001.

We observed an increase of 0.16 points in the rate of hospital infections in Spain, going from 1.2 in 2003 to 1.3 in 2013, with an increasing trend (APC = 0.0003, 95%CI 0.0002–0.0004) (Table 3).

**Table 3:** Indicator: hospital infection rate in descending order. Spain, 2003–2014.

AC	2003	2014	APC	95%CI	Trend
Canary Islands	1.2	1.7	0.0012	0.0003–0.0014	Increasing^a^
Balearic Islands	1.0	1.5	0.0011	0.0008–0.0014	Increasing^b^
Galicia	1.1	1.3	0.0008	-0.001–0.0016	Increasing^a^
Andalusia	1.1	1.5	0.0009	0.0006–0.0013	Increasing^b^
Valencia	0.6	0.8	0.0006	0.0004–0.0008	Increasing^b^
Murcia	0.8	0.9	0.0003	0.0001–0.0006	Increasing^a^
Spain	1.2	1.2	0.0003	0.0002–0.0004	Increasing^b^
Asturias	1.4	1.4	0.0002	-0.001–0.0015	Static
Catalonia	1.2	1.2	0.0001	-0.0046–0.0009	Static
La Mancha	1.1	1.1	0.0001	-0.0002–0.0004	Static
Basque Country	1.1	1.2	0.0001	-0.0002–0.0004	Static
Madrid	1.4	1.4	< 0.0001	-0.0003–0.0004	Static
Navarre	0.8	0.7	< 0.0001	-0.0003–0.0003	Static
La Rioja	1.2	1.0	-0.0002	-0.0008–0.0004	Static
Ceuta	1.1	0.8	-0.0003	-0.0011–0.0006	Static
Cantabria	2.1	1.9	-0.0004	-0.0008– > -0.0001	Decreasing^a^
Extremadura	1.0	0.8	-0.0005	-0.0007– -0.0002	Decreasing^b^
Aragon	1.5	1.2	-0.0006	-0.0008– -0.0004	Decreasing^b^
Castile and León	1.6	1.3	-0.0006	-0.0838– -0.0399	Decreasing^b^
Melilla	1.3	0.5	-0.0016	-0.0019– -0.0014	Decreasing^b^

AC: autonomous community; APC: annual percent change

a Significance level p < 0.05.

b Significance level p < 0.001.

The increase in in-hospital mortality in Spain was 0.3 points, going from 3.9 to 4.2 per 100 hospital discharges in the period studied, with an increasing trend (APC = 0.0008, 95%CI 0.0006–0.0011) (Table 4).

**Table 4:** Indicator: global in-hospital mortality per 100 hospital discharges in descending order. Spain, 2003–2014.

AC	2003	2014	APC	95%CI	Trend
La Mancha	4.2	4.7	0.0037	< 0.0001–0.0067	Increasing^a^
Extremadura	3.8	4.8	0.0029	0.0019–0.0039	Increasing^b^
Navarre	3.2	4.6	0.0026	0.0008–0.0048	Increasing^a^
Galicia	4.5	5.3	0.0025	0.0019–0.0031	Increasing^b^
Canary Islands	3.7	4.8	0.0023	0.0015–0.0033	Increasing^b^
Andalusia	4.1	4.7	0.0021	0.0018–0.0024	Increasing^b^
Castile and León	4.1	5.0	0.0018	0.0012–0.0023	Increasing^b^
Ceuta	3.4	3.7	0.0009	< 0.0001–0.0018	Increasing^a^
Spain	3.9	4.2	0.0008	0.0006–0.0011	Increasing^b^
Asturias	4.5	5.2	0.0008	-0.0003–0.0621	Static
Basque Country	3.9	4.0	0.0008	-0.0003–0.0002	Static
Balearic Islands	3.2	3.5	0.0007	0.0003–0.001	Increasing^a^
Murcia	3.5	3.6	0.0005	-0.0003–0.0015	Static
Valencia	3.9	4.0	0.0004	0.0001–0.0008	Increasing^a^
Aragon	4.2	4.4	0.0001	-0.0002–0.0005	Static
Catalonia	3.6	3.2	-0.0004	-0.0008–0.0001	Static
Melilla	3.8	4.0	-0.0005	-0.0012–0.0003	Static
Cantabria	5.1	4.7	-0.0006	-0.0012–0.0001	Static
Madrid	4.0	3.9	-0.0006	-0.0064– -0.0002	Decreasing^a^
La Rioja	3.7	3.3	-0.0007	-0.0012– -0.0002	Decreasing^a^

AC: autonomous community; APC: annual percent change

a Significance level p < 0.05.

b Significance level p < 0.001.

## DISCUSSION

The data show an increasing trend in the indicators of expenditure on SC services, nursing and medical staff in SC per 1,000 inhabitants, working operating rooms per 1,000 inhabitants, surgical interventions per 1,000 inhabitants, rate of hospital infections, and in-hospital mortality for every 100 hospital discharges. Nevertheless, we observed a decreasing trend in the indicator of average duration. Similarly, we found a static trend in the indicators of patients waiting for specialized care appointments and patients waiting for non-urgent surgical interventions per 1,000 inhabitants.

Health expenditure on SC has been increasing in most Autonomous Communities. However, high health expenditure to address these problems is not a sufficient condition to ensure good public health or good quality of the services. Regardless of a greater or lesser expenditure, the services provided need to have greater efficiency^13^.

This increase in health expenditure on SC can be related to the demographic change and technological development^14^, although it clearly contradicts the principles of health promotion and disease prevention of the General Health Law^2^. A good strategy to foster healthy living habits and prevent disease is the development of primary care^15^. This would avoid unnecessary demand for SC. We observed an increase of 9.5 points in health expenditure on SC between 2003 and 2014 when compared to primary care expenditure, which went from 13.9% in 2003 to 13.4% in 2014^11^, with a decrease of 0.5 percentage points. Thus, it seems appropriate to analyze whether the increase in the frequency and resources at the SC level presupposes a decrease of resources destined to primary care. This could strengthen the role of primary care in the SNS to reorient the current centrist hospital approach of the SNS^16^.

On the other hand, there are many factors that can influence the increase of these costs, such as the use of resources and clinical variability, the low integration between care levels, the adoption of new technologies, or the increase in aging and less health habits that require greater care^13^ ^,^ ^17^ ^,^ ^18^. All these factors imply a challenge for the SNS, as the need to put into operation new health management strategies is evident. Competency management can be one of these strategies, with a greater involvement of professionals, citizens, and administrations to address this problem, since it ensures the sustainability of the health system in relation to the costs related and adapted to the needs of society^19^.

The increase in SC expenditure contrasts with key indicators that assess the quality of care, such as the increase in the rate of hospital infections and in-hospital mortality. These aspects could be related to the inadequate prevention of nosocomial infections, the high workload of professionals, the increase in the number of older patients whose health condition is more severe, or the increase in the multiresistance of some microorganisms. More studies are needed to discern these causes, especially to determine if these infections could be avoided and to establish measures such as increasing the competence of professionals for their prevention^20^ ^–^ ^23^.

Another indicator that measures the situation of the SC and that contrasts with those mentioned previously is the average duration, used to determine the level of hospital efficiency. We verified a decrease of this indicator in all AC, except Ceuta, La Mancha, Castile and León, and Murcia which show a static trend. This decrease can be justified by advances in the diagnosis and treatment of diseases and technological innovations^24^. The SC improves when the hospital stay is prolonged, which is a worldwide concern for the negative effects that it entails, such as: increased costs, saturation of services, and decreased quality of care regarding the risks of adverse events^25^. However, the rate of nosocomial infections and the rate of in-hospital mortality in Spain kept increasing trends in the period.

Despite showing an increasing trend in Spain and some AC in the temporary series, the number of professionals in both groups decreased in Spain and in most of the AC in 2012, and it gradually recovered in some cases. This likely comes from the onset of the economic crisis and the consequences of the cuts, which affected each area with different intensity and pace of application, increasing inequality and calling into question the cohesion of the public health system^26^. In addition, the nurse-patient rate is low when compared to the average of the countries of the Organization for Economic Co-operation and Development (OECD)^27^ (5.2 per 1,000 inhabitants compared with the 8.8 recommended by the organization). Spain is the country number 28 of 34, of all OECD countries. However, the physician-patient rate (3.8) is above that recommended by the OECD (3.2).

According to Price Waterhouse Coopers^28^, not every strategy is valid to ensure the sustainability of a health system in a context of economic crisis. Measures aimed at reducing expenditure, rather than improving efficiency, should not be adopted without a long-term view of the consequences that may be incurred. Economic cuts and inefficient management increasingly affect professionals and consequently the quality of care, forgetting that the goal of the SNS is to improve the well-being and satisfaction of users^19^ ^,^ ^29^ ^,^ ^30^.

Despite the increasing trend of working operating rooms and surgical interventions per 1,000 inhabitants, along with the increasing trend of nursing and medical staff and increased SC expenditure, we observed a static trend in patients waiting for non-urgent surgical interventions in the study period. This is another example of the inadequate use of resources in SC and it manifests the problems of sustainability and the possibility of improving resource management^31^ ^,^ ^32^. More attention should be given to how money is spent before choosing to increase the funding of a service. However, the data removed by the AC are missing. Therefore, we cannot relate if this increase is because of those AC that did not increase their resources^26^.

On the other hand, we need to consider a series of limitations, such as the lack of data from the AC for some indicators (for example, surgical waiting lists)^26^, which require us to consider this analysis with caution and see it as approximate. Research studies need to be developed with correlated methodological projects that allow the further study on the causes that have led to the decrease of most of the indicators of SC during the studied period. This would allow the establishment of measures to meet the needs of the population, taking into account the criteria of quality of care, equity, and cost-benefit of interventions. A study of interrupted time series of the indicators of the SNS would allow the analysis of the impact of the Royal Decree-Law 16/2012, on urgent measures, to ensure the sustainability of the SNS and improve the quality and safety of its care^8^.

The analysis of the evolution of the Key Indicators of the National Health System of SC shows how the principles are not being complied with for the equity and quality of care offered. The main causes may be increased aging, technological development, and inadequate strategies being taken to reduce health costs without bearing in mind the consequences that can be produced in the medium and long term. Despite the increase in health expenditure on SC, we observed a worsening in key health quality indicators.
